# Short-form video usage and cognitive function among rural older adults in northern China: a cross-sectional study

**DOI:** 10.3389/fpubh.2026.1873982

**Published:** 2026-07-14

**Authors:** Hanxuan Fu, Min Fei, Ruiying He, Siming Wu, Fei Wang

**Affiliations:** 1 Department of Clinical Medicine, Changzhi Medical College, Changzhi, Shanxi, China; 2 Department of Neurology, Yuncheng Central Hospital Affiliated to Shanxi Medical University, Yuncheng, Shanxi, China

**Keywords:** cognitive function, digital media, northern China, rural older adults, short-form video

## Abstract

**Introduction:**

The increasing popularity of short-form video platforms, particularly among older adults, prompts questions about their impact on cognitive health. This study examines the association between short-form video use and cognitive function among rural older adults in northern China, a demographic often overlooked in digital media research.

**Methods:**

A cross-sectional study was conducted in Xia County, Shanxi Province, China, from April to June 2024. Community-dwelling adults aged 65 years and older were selected using a two-stage cluster sampling method. Cognitive function was evaluated via the Montreal Cognitive Assessment (MoCA) scale, assessing domains such as memory and attention. Data were collected via face–to–face interviews, and multivariate linear regression models were used to analyze the associations between short-form video use and cognitive function, controlling for potential confounders.

**Results:**

Among the 1,501 participants, 818 (54.4%) were regular short-form video users. After adjusting for confounders, multivariate analysis revealed a positive association between short-form video use and cognitive scores. Subgroup analyses indicated stronger associations among individuals who were less engaged in other cognitive activities, such as reading or watching TV. Additionally, users who viewed short-form videos for information-seeking, social connection, and educational purposes, or those who actively interacted with the content, exhibited even higher cognitive performance.

**Conclusions:**

Short-form video usage is prevalent among rural Chinese older adults and is positively associated with cognitive performance, particularly for those with limited access to alternative activities, although causality cannot be inferred due to the cross-sectional design. This study highlights an understudied association and emphasizes the need to include marginalized rural populations in digital health and cognitive aging research.

## 1 Introduction

As the prevalence of cognitive impairment continues to rise, cognitive decline has become a substantial global challenge among aging populations (1). Currently, it is estimated that 50 million people worldwide live with various forms of cognitive impairment, a figure projected to triple by 2050 (2). The ramifications of these conditions extend well beyond the affected individuals, imposing significant strain on families, communities, and healthcare systems (2). In China, the challenges associated with population aging and cognitive impairment are particularly acute. By the end of 2024, individuals aged 60 and older had accounted for 22.0% of the national population, with dementia prevalence in this demographic estimated at 6.04% (3, 4). Efforts to address these challenges have focused on managing modifiable risk factors, such as hypertension, diet, and smoking (5). Additionally, identifying and leveraging new modifiable risk factors is of paramount importance.

Short-form videos, a contemporary phenomenon within the digital media landscape, refer to condensed audiovisual content of brief duration, typically under 1 min. Available on platforms such as TikTok and Kuaishou, this genre has gained immense popularity due to its brevity, provision of instant gratification, suitability for mobile consumption and facilitation of grassroots content creation, making it appealing across all age groups, including older adults (6). By June 2024, China alone boasted a staggering 1.05 billion short-form video users, with 8.7% aged 60 years and older (7). Notably, approximately 35% of these senior users reported more than 1 h of daily use (8), suggesting that short-form videos have become an integral part of their daily routines.

Despite this widespread and increasing engagement with short-form videos among older adults, research on their cognitive impact—especially in rural areas—remains limited. Existing research on digital technology and older adults' cognition often focuses on broader internet use, social media, or smartphone activities, which have demonstrated potential benefits for cognitive function and social wellbeing (9–13). In contrast, studies on short-form videos center predominantly on younger populations, highlighting concerns related to attention spans, addictive usage patterns, and mental health impacts. For example, research on college students has found that frequent short-form video consumption is negatively associated with sustained attention and working memory (14, 15), while excessive use may contribute to sleep disturbances and emotional distress (16).

Addressing this gap requires special attention to the unique context of older adults in rural China, where structural, technological, and social disparities profoundly shape cognitive aging trajectories (17). Structurally, rural older adults experience compounded disadvantages, including poorer healthcare access, limited availability of preventive health services, inadequate chronic disease management, physically demanding occupational histories, less favorable health-related lifestyles, and poorer dietary conditions—all of which contribute to elevated risks of vascular and metabolic conditions that accelerate cognitive decline (18, 19). Coupled with lower formal education levels and fewer opportunities for cognitive stimulation through traditional media such as books, movies, or games, these factors collectively result in lower baseline cognitive reserve (20).

Technologically, this structural marginalization directly dictates a stark “digital divide” fueled by a deficit in “digital endowment”—the accumulated digital literacy and capability required to navigate modern technology (21). In rural China, the primary level of the digital divide (access to hardware and connectivity) has been partially bridged by smartphones, yet the secondary level (capability and usage digital divides) remains a formidable barrier (22–24). Such barrier not only constrains rural older adults' willingness and ability to engage with emerging media like short-form videos, but also shapes the potential health returns from technology use. Furthermore, rural older adults often face higher risks of social isolation (17), as younger family members often migrate to urban areas for work. Considering these unique factors, short-form videos may represent a viable tool for cognitive support among rural older adults. Specifically, the concise content and intuitive design of these platforms (8) may provide a novel and low-barrier source of mental stimulation (25), while the inherent participatory culture may foster social connection by facilitating interaction with family and communities, potentially buffering the neurocognitive detriments of loneliness (26). Conversely, rural older adults remain particularly vulnerable to the downsides of these platforms. For example, the fragmented, algorithm-driven content feeds can induce cognitive overload and attention deficits (14, 27–29), while a propensity for passive consumption may displace more active, cognitively stimulating engagement (8, 30, 31). Furthermore, limited digital endowment renders rural older adults highly susceptible to predatory misinformation and cyber fraud, which commercial algorithms systematically exploit (32). Therefore, empirical evidence is needed to determine whether short-form video use serves as a cognitive asset or a liability within this multifaceted structural-technological-social context.

Given this complex landscape, this study aimed to: (1) document the prevalence and usage patterns of short-form videos among rural older adults in northern China; and (2) assess their correlation with cognitive performance.

## 2 Materials and methods

### 2.1 Study design and participants

This cross-sectional study was conducted in Xia County, located in the Yuncheng Prefecture of southern Shanxi Province, northern China, from April to June 2024. Xia County is a predominantly rural area with a population of approximately 280,000, characterized by an agricultural economy and a high proportion of older adults (16.3% aged 65 years and older, exceeding the national average of 13.5%) (33). This region was selected for its representativeness of rural northern China and logistical feasibility for conducting household surveys.

Participants were community-dwelling rural adults aged 65 years and older, recruited via a two-stage cluster sampling method. In the first stage, Yaofeng Town was selected based on its demographic and economic profile. In the second stage, 15 villages within the town were randomly chosen using a random number generator. A comprehensive sampling frame of registered residents meeting the age criteria was then established through official administrative records. With the assistance of local authorities and community health workers, trained researchers conducted home visits to screen for eligibility and obtain informed consent.

Study inclusion criteria comprised: (1) age 65 years or older; (2) registered residence in the target area for at least 1 year prior to the survey; and (3) ability to provide informed consent and complete the survey. Exclusion criteria were: (1) refusal to participate; and (2) severe physical or mental disorders that precluded completion of the assessments. Such disorders were identified through self-reported medical history, caregiver reports, or investigator observations during initial screening. Of 1,623 individuals approached, 1,501 (92.5%) participated, with 122 excluded due to severe health issues (*n* = 74) or refusal (*n* = 48).

### 2.2 Operational procedures and data collection

A comprehensive questionnaire was structured to capture demographic characteristics, lifestyle habits, medical history, and detailed patterns of short-form video use (Supplementary file S1). To ensure linguistic clarity and content relevance, the instrument underwent pilot testing with a representative subset of the target population. Cognitive function was evaluated using a localized Chinese adaptation of the Montreal Cognitive Assessment (MoCA) scale. This adaptation was chosen based on its extensive validation and widespread application among older adults in China (34, 35). For participants with low literacy, simplified oral instructions and contextual clarifications were provided for challenging tasks (e.g., abstraction, language), and culturally appropriate examples were offered to enhance comprehension without altering the standard scoring procedure.

Prior to data collection, the investigators underwent standardized training to ensure strict adherence to the study protocol and ethical guidelines. The study protocol was approved by the Institutional Review Board of Yuncheng Central Hospital. All participants received a comprehensive briefing regarding the study objectives, procedures, and potential risks and benefits before providing informed consent. Data collection was conducted via face-to-face interviews in a private home setting to ensure comfort and confidentiality. To ensure data completeness and quality, interviewers verified the questionnaires for any omissions or logical inconsistencies immediately after each interview. Any missing information was clarified and completed on-site. Consequently, the final dataset contained no missing values, and no imputation methods were required.

### 2.3 Short-form video engagement

Short-form video consumption was operationalized as active engagement with platforms such as Douyin, TikTok, and Kuaishou for a minimum of 30 min daily. The metrics of short-form video engagement included duration, frequency, motives, and interaction depth. These measures were adapted from prior digital media studies (9, 10) and refined through pilot testing to ensure clarity and response accuracy.

Usage duration was defined as the number of years since initial engagement with short-form video platforms, while usage frequency was measured by average daily viewing time. Motives for use were categorized into six domains: leisure, information seeking, social connection, education, documentation, and monetization. Usage depth was operationalized by the diversity of interactive behaviors (e.g., liking, commenting, sharing, and uploading) to differentiate between passive consumption and active participation. These metrics were captured using a semi-structured approach: participants first responded to open-ended inquiries (e.g., “How many years have you been engaging with short-form videos?” and “What are your main reasons for engaging with short-form videos?”). For those unable to provide spontaneous answers, standardized multiple-choice prompts were administered to ensure data completeness and minimize recall bias.

### 2.4 Cognitive function

Cognitive function was quantified using the MoCA total score alongside five specific cognitive domains derived from the scale: memory (5 points, from delayed recall), orientation (6 points, from orientation), executive function (5 points, from visuospatial/executive), attention (6 points, from attention) and language (8 points, combined from the naming, language and abstraction). This multi-domain approach allowed for a more granular analysis of the cognitive correlates of short-form video use.

### 2.5 Covariates

To control for potential confounding, three categories of covariates were included in the analysis: sociodemographic characteristics (age, sex, educational level, marital status, occupation, number of cohabitants, and monthly income), health-related factors (history of hypertension, diabetes mellitus, stroke, and heart disease), and lifestyle habits (smoking status, alcohol use, sleep complaints, physical activity levels, and engagement in recreational activities such as reading, board games, or watching TV). These covariates were incorporated to mitigate potential confounding effects on both the exposure variables (short-form video use) and the outcome variables (cognitive function).

### 2.6 Statistical analysis

Data were collected and managed using the WPS Office Smart Form platform (Kingsoft Corporation, Beijing, China). Statistical descriptions and analyses were conducted via R version 4.3.0 (R Foundation for Statistical Computing, Vienna, Austria). Baseline characteristics and cognitive scores were compared between short-form video users and non-users. Quantitative data were presented as means ± standard deviations or medians with interquartile ranges (IQRs), contingent upon the data distribution. To evaluate the association between short-form video engagement and cognitive performance, multivariate linear regression models were employed. These models adjusted for the aforementioned confounders to estimate the independent association between usage patterns and both total MoCA scores and specific cognitive domains. Ordinal covariates—such as age group, education, income, and physical activity—were treated as categorical variables and entered into the models as dummy variables. For all ordinary least squares (OLS) regression models, assumptions of multicollinearity [generalized variance inflation factor (GVIF)], normality of residuals (Q-Q plots and Shapiro–Wilk tests), and homoscedasticity (Breusch–Pagan tests) were examined. Influential cases were assessed using Cook's distance. Given the bounded and discrete nature of MoCA subscores, sensitivity analyses using ordinal logistic regression were conducted to verify the robustness of the OLS estimates.

Exploratory analyses were performed across subgroups based on age, sex, income level, and lifestyle habits to gain a granular perspective of the associations across different demographics. Moreover, within the user cohort, the associations between different motives and depths of usage with cognitive scores were analyzed using multivariate linear regression models. This comprehensive approach ensures the robustness of the study's findings by accounting for a broad spectrum of potential influencing factors. Statistical significance was defined as a two-tailed *p* < 0.05.

## 3 Results

### 3.1 Overall characteristics

Among the 1,501 participants, 818 (54.4%) reported regular consumption of short-form videos. Baseline comparisons between users and non-users revealed significant disparities across multiple dimensions. Specifically, short-form video users were more likely to be male, younger, have higher socioeconomic status, have a partner, be more physically active, smoke, consume alcohol, and play board/card games. Cognitive assessments revealed that the user group had significantly higher scores on the MoCA and all five subdomains. Detailed characteristics according to short-form video use are presented in Table 1.

**Table 1:** Characteristics of the study population by short-form video use.

Variables	Total (*n* = 1,501)	Short-form video use		Statistic	*p-*Value
		**No (*n* = 683)**	**Yes (*n* = 818)**		
Sex, *n* (%)				*V* = 0.070	0.007
Female	778 (51.83)	380 (55.64)	398 (48.66)		
Male	723 (48.17)	303 (44.36)	420 (51.34)		
Age, *n* (%)				*V* = 0.397	< 0.001
65–69	495 (32.98)	119 (17.42)	376 (45.97)		
70–74	407 (27.12)	165 (24.16)	242 (29.58)		
75–79	339 (22.58)	191 (27.96)	148 (18.09)		
≥80	260 (17.32)	208 (30.45)	52 (6.36)		
Marital status, *n* (%)				*V* = 0.177	< 0.001
Married	1,173 (78.15)	479 (70.13)	694 (84.84)		
Divorced/Widowed/Unmarried	328 (21.85)	204 (29.87)	124 (15.16)		
Education level, *n* (%)				*V* = 0.296	< 0.001
Uneducated	237 (15.79)	172 (25.18)	65 (7.95)		
Primary school	592 (39.44)	294 (43.05)	298 (36.43)		
Junior high	500 (33.31)	178 (26.06)	322 (39.36)		
High school	154 (10.26)	37 (5.42)	117 (14.30)		
College and above	18 (1.20)	2 (0.29)	16 (1.96)		
Occupation, *n* (%)				*V* = 0.124	< 0.001
Farming	1,274 (84.88)	613 (89.75)	661 (80.81)		
Service	155 (10.33)	48 (7.03)	107 (13.08)		
Manual labor	72 (4.80)	22 (3.22)	50 (6.11)		
Monthly income in CNY, *n* (%)				*V* = 0.170	< 0.001
< 200	723 (48.17)	376 (55.05)	347 (42.42)		
200–500	306 (20.39)	125 (18.30)	181 (22.13)		
501–1,000	102 (6.80)	24 (3.51)	78 (9.54)		
1,001–2,000	218 (14.52)	106 (15.52)	112 (13.69)		
>2,000	152 (10.13)	52 (7.61)	100 (12.22)		
Number of cohabitants, *n* (%)				*V* = 0.124	< 0.001
0	189 (12.59)	115 (16.84)	74 (9.05)		
1	822 (54.76)	342 (50.07)	480 (58.68)		
≥2	490 (32.64)	226 (33.09)	264 (32.27)		
Physical activity^a^, *n* (%)				*V* = 0.140	< 0.001
Low	330 (21.99)	188 (27.53)	142 (17.36)		
Moderate	598 (39.84)	275 (40.26)	323 (39.49)		
High	573 (38.17)	220 (32.21)	353 (43.15)		
Sleep complaint, *n* (%)				*V* = 0.014	0.589
No	1,209 (80.55)	546 (79.94)	663 (81.05)		
Yes	292 (19.45)	137 (20.06)	155 (18.95)		
Smoking^b^, *n* (%)				*V* = 0.073	0.004
No	1,128 (75.15)	537 (78.62)	591 (72.25)		
Yes	373 (24.85)	146 (21.38)	227 (27.75)		
Alcohol use^c^, *n* (%)				*V* = 0.052	0.046
No	1,391 (92.67)	643 (94.14)	748 (91.44)		
Yes	110 (7.33)	40 (5.86)	70 (8.56)		
Reading^d^, *n* (%)				*V* = 0.037	0.155
No	1,372 (91.41)	632 (92.53)	740 (90.46)		
Yes	129 (8.59)	51 (7.47)	78 (9.54)		
Board/Card games^e^, *n* (%)				*V* = 0.080	0.002
No	1,121 (74.68)	536 (78.48)	585 (71.52)		
Yes	380 (25.32)	147 (21.52)	233 (28.48)		
TV watching^f^, *n* (%)				*V* = 0.215	< 0.001
No	331 (22.05)	84 (12.30)	247 (30.20)		
Yes	1,170 (77.95)	599 (87.70)	571 (69.80)		
Hypertension, *n* (%)				*V* = 0.087	< 0.001
No	853 (56.83)	356 (52.12)	497 (60.76)		
Yes	648 (43.17)	327 (47.88)	321 (39.24)		
Diabetes, *n* (%)				*V* = 0.023	0.373
No	1,354 (90.21)	611 (89.46)	743 (90.83)		
Yes	147 (9.79)	72 (10.54)	75 (9.17)		
History of stroke, *n* (%)				*V* = 0.079	0.002
No	1,267 (84.41)	555 (81.26)	712 (87.04)		
Yes	234 (15.59)	128 (18.74)	106 (12.96)		
Heart disease, *n* (%)				*V* = 0.032	0.214
No	1,314 (87.54)	590 (86.38)	724 (88.51)		
Yes	187 (12.46)	93 (13.62)	94 (11.49)		
MoCA score, Mean ± SD	16.48 ± 5.82	13.46 ± 5.48	18.99 ± 4.80	*t* = −20.58	< 0.001
Subdomain score, *M* (Q_1_, Q_3_)					
Memory	2.00 (1.00, 3.00)	1.00 (1.00, 2.00)	2.00 (2.00, 3.00)	*Z* = −14.89	< 0.001
Orientation	5.00 (4.00, 6.00)	5.00 (3.00, 6.00)	6.00 (5.00, 6.00)	*Z* = −10.36	< 0.001
Executive	1.00 (0.00, 3.00)	0.00 (0.00, 1.00)	2.00 (1.00, 3.00)	*Z* = −14.04	< 0.001
Attention	5.00 (3.00, 6.00)	4.00 (2.00, 5.00)	6.00 (4.00, 7.00)	*Z* = −17.31	< 0.001
Language	4.00 (3.00, 5.00)	3.00 (2.00, 4.00)	5.00 (3.00, 6.00)	*Z* = −16.74	< 0.001

t: t-test; Z: Mann–Whitney test; V, Cramér's V; CNY, Chinese Yuan; SD, standard deviation; M, Median; Q1, 1st Quartile; Q3, 3rd Quartile. ^a^Physical activity levels are defined as: low: < 150 min/week of moderate-intensity physical activity or the equivalent amount of vigorous-intensity physical activity. Moderate: 150–300 min/week of moderate-intensity physical activity or equivalent. High: >300 min/week of moderate-intensity physical activity or equivalent. ^b^Smoking was defined as having smoked at least 400 cigarettes. ^c^Alcohol use was defined as drinking at least 0.1 drink per day for 1 year or more, with one drink equal to 10 g pure alcohol. ^d^Reading was defined as reading at least 1 h/week. ^e^Board/Card game use was defined as playing poker/mahjong/chess at least 1 h/week. ^f^TV watching was defined as watching TV at least 0.5 h/day.

### 3.2 Associations between short-form video use and cognitive function

After adjusting for confounding factors, multivariate linear regression analysis revealed that short-form video users had higher scores on the MoCA (β = 3.01, 95% CI = 2.53–3.49, standardized β = 0.257; partial *R*^2^ = 0.093), and all five subdomains than non-users (Table 2, Supplementary Table S2), indicating a positive association between short-form video consumption and better cognitive performance. The adjusted *R*^2^ values for the fully adjusted models ranged from 0.190 for orientation to 0.499 for the MoCA total score, indicating moderate explanatory power. All adjusted GVIFs were below 2, indicating no severe multicollinearity. Breusch–Pagan tests suggested heteroscedasticity in several models; therefore, heteroscedasticity-consistent HC3 robust standard errors were applied. Both the HC3-corrected estimates and sensitivity analyses using ordinal logistic regression yielded consistent directions and significance levels for the predictors, confirming the robustness of the OLS findings. Detailed model statistics, residual diagnostics, and sensitivity analysis results are provided in Supplementary Table S2.

**Table 2:** Associations between short-form video use and cognitive function.

Short-form video use	Variable	Model 1	Model 2	Model 3	Model 4	Model 5
		β (95%CI)	β (95%CI)	β (95%CI)	β (95%CI)	β (95%CI)
No		0.00 (Reference)	0.00 (Reference)	0.00 (Reference)	0.00 (Reference)	0.00 (Reference)
Yes	MoCA	5.53 (5.01–6.05)^*^	4.23 (3.70–4.76)^*^	3.16 (2.67–3.64)^*^	3.04 (2.57–3.52)^*^	3.01 (2.53–3.49)^*^
	Memory	0.87 (0.76–0.98)^*^	0.60 (0.49–0.72)^*^	0.48 (0.37–0.60)^*^	0.46 (0.35–0.58)^*^	0.46 (0.35–0.58)^*^
	Orientation	0.76 (0.63–0.90)^*^	0.60 (0.46–0.74)^*^	0.44 (0.30–0.59)^*^	0.43 (0.28–0.57)^*^	0.43 (0.29–0.57)^*^
	Executive	0.96 (0.82–1.09)^*^	0.74 (0.60–0.88)^*^	0.50 (0.37–0.63)^*^	0.48 (0.35–0.61)^*^	0.47 (0.34–0.60)^*^
	Attention	1.91 (1.72–2.10)^*^	1.47 (1.27–1.66)^*^	1.10 (0.92–1.28)^*^	1.07 (0.89–1.25)^*^	1.05 (0.87–1.23)^*^
	Language	1.51 (1.35–1.67)^*^	1.20 (1.04–1.37)^*^	0.92 (0.76–1.08)^*^	0.89 (0.73–1.05)^*^	0.86 (0.70–1.02)^*^

Multivariate linear regression models. ^*^p < 0.001. CI, confidence interval. Model 1: Crude. Model 2: Adjust: sex, age, marital status, number of cohabitants. Model 3: Adjust: sex, age, marital status, education level, occupation, monthly income, number of cohabitants. Model 4: Adjust: sex, age, marital status, education level, occupation, monthly income, number of cohabitants, smoking, alcohol use, physical activity, reading, board/card games. Model 5: Adjust: sex, age, marital status, education level, occupation, monthly income, number of cohabitants, smoking, alcohol use, physical activity, reading, board/card games, TV watching, sleep complaint, hypertension, diabetes, stroke, heart disease.

### 3.3 Subgroup analyses

**Figure 1:**
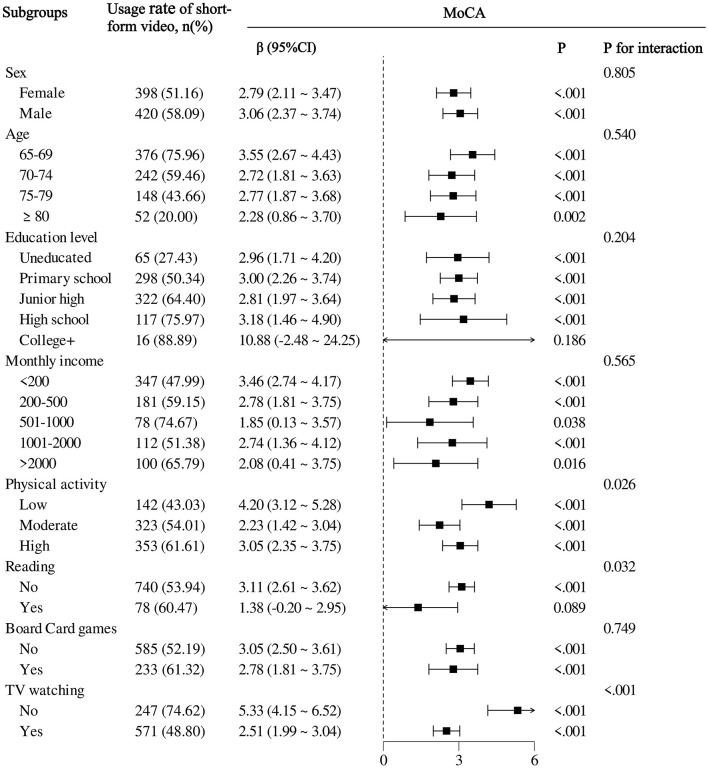
Associations between short-form video use and MoCA scores among exploratory subgroups. After adjusting for sex, age, education level, marital status, monthly income, physical activity, reading, board/card games, TV watching, and history of stroke as confounding factors, a multivariate linear regression model was employed to explore the effects of short-form video use on cognitive performance within distinct subgroups. Additionally, interaction effects were examined within the model.

The usage rates of short-form videos varied across different subgroups (Figure 1). Males exhibited a higher usage rate (58.09%) than females (51.16%). Additionally, short-form videos were more popular among younger, more educated individuals and those who were more physically or cognitively active (e.g., reading, playing board/card games). Conversely, short-form video usage was substantially lower among regular television viewers compared to non-viewers.

Subgroup analyses further demonstrated that the associations between short-form video use and higher cognitive scores were consistent across different sociodemographic groups (e.g., age, sex, education level, and monthly income). However, when stratified by lifestyle habits, these positive associations were significantly more pronounced among older adults who did not regularly exercise, read, or watch TV (*p* for interaction < 0.05; Figure 1).

### 3.4 Characteristics of short-form video consumption in the user group

Within the user cohort (*n* = 818), the median daily use of short-form videos was 2.0 h (IQR: 1.0–3.0 h). The median duration of use was 3 years (IQR: 2–5 years). The primary motive for use was leisure (97.80%), followed by information seeking (42.54%), social connection (25.92%), education (15.28%), documentation purposes (10.51%), and monetization (2.44%). Among the 818 users, 354 (43.3%) used short-form videos solely for leisure purposes.

**Figure 2:**
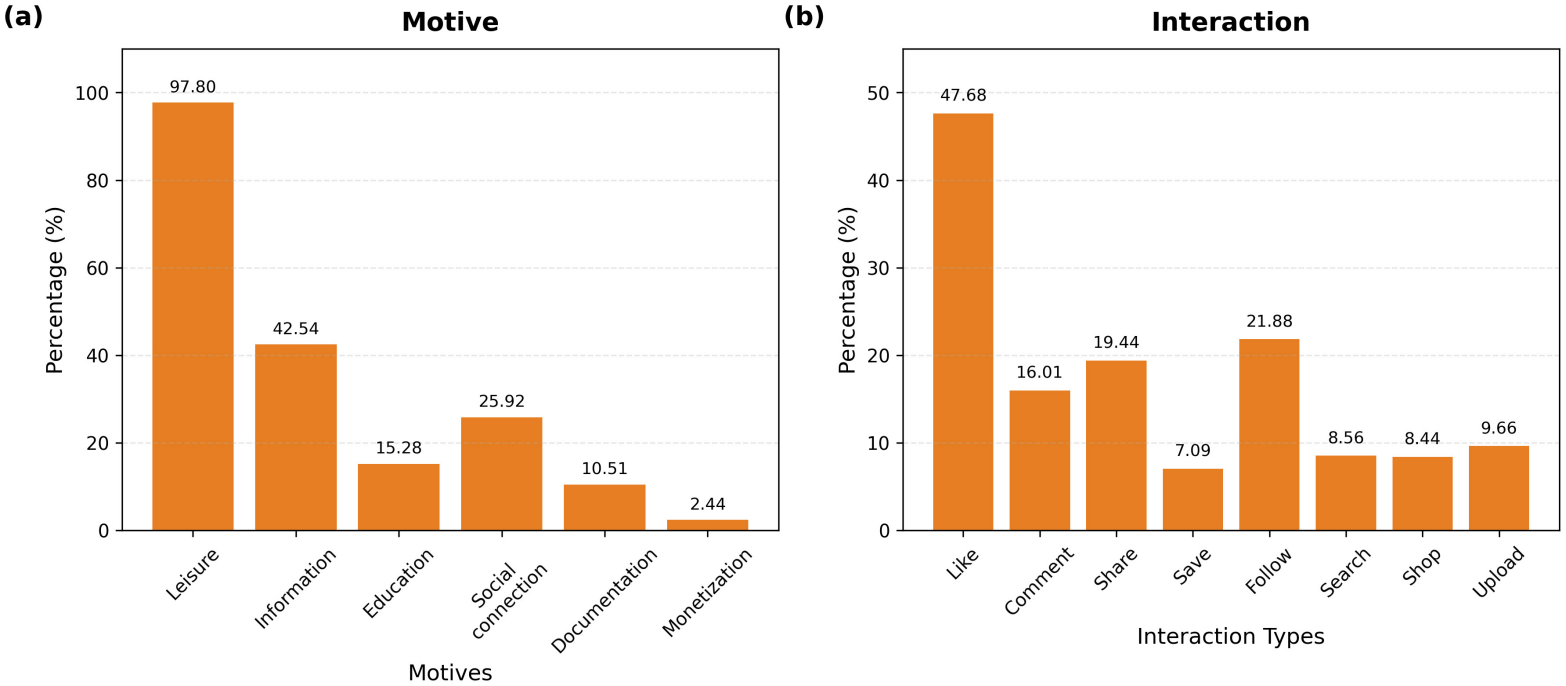
Distribution of motives and interactive behaviors among short-form video users.

Regarding engagement depth, a majority of users (50.3%) passively viewed and swiped through the content without engaging in interactive behaviors such as liking, commenting or sharing. Only 11.0% of users engaged in five or more types of such behaviors. Figure 2 presents the distribution of these specific user interactions. The median number of interaction types was 0 (IQR: 0–2).

### 3.5 Associations between motives, interactive behaviors and cognitive function among short-form video users

When the analysis was restricted to short-form video users, multivariate linear regression models adjusted for potential confounders revealed that use for leisure was associated with lower executive and overall cognitive performance. Conversely, users who engaged for information seeking, education, or social connection motives had significantly higher scores on the MoCA and all five subdomains than those without such motives (Supplementary Table S5).

Furthermore, a positive linear trend was identified between the diversity of interactive behaviors and cognitive scores. Users who actively participated through various interactions (e.g., liking, sharing) demonstrated significantly higher performance on the MoCA and its five subdomains than those without such interactions (Supplementary Table S1).

## 4 Discussion

Our findings demonstrate that short-form video use is highly prevalent among older adults in rural China and is significantly associated with better cognitive performance. This positive association was particularly evident among those with lower levels of other physical or cognitive activities, suggesting a potential compensatory role. While most participants used these videos primarily for leisure and in a passive manner, more active engagement—such as seeking information or interacting with content—was linked to even higher cognitive performance.

The high prevalence of short-form video consumption observed herein diverges from earlier regional investigations (10, 23, 36), but aligns with global trends toward pervasive digital media uptake among older populations (37). Such widespread penetration reflects the deep embedding of short-video platforms into the daily lives of rural older adults, signaling that the primary digital divide (hardware access) has substantially narrowed in rural Northern China. However, our granular analysis reveals that this digital adoption is uneven, with more frequent usage concentrated among younger males and individuals with higher socioeconomic status. These demographic disparities align with existing literature (38, 39) but critically illustrate the persistence of the secondary digital divide (the usage and capability gap) within this population. Rather than reflecting mere variations in individual cognitive flexibility, these disparities are deeply rooted in unequal livelihood capital and digital endowments (21). Collectively, these patterns indicate that short-form videos can penetrate disadvantaged rural older groups whose offline cognitive and social resources are structurally constrained, while also mirroring digital inequalities across the rural aging population.

The mean MoCA scores in our sample of rural older adults were significantly lower than those reported for non-rural older populations (40–44). This baseline disparity underscores the pervasive rural-urban health inequities, rooted in structural deficits in healthcare, unhealthy lifestyles, and socioeconomic deprivation (18). Despite this, we observed a positive association between short-form video consumption and higher cognitive performance. This positive correlation can be explained from two perspectives. First, reverse causation and selection bias provide a highly plausible explanation. Older adults with better baseline cognitive abilities may simply be more likely to adopt and regularly engage with short-form video platforms in the first place. Despite the relatively intuitive design of these platforms, navigating digital interfaces, understanding content, and engaging regularly still requires sufficient cognitive capacity and digital skills. This selection mechanism is well-documented in the broader literature on technology adoption and aging (45–48). Second, it remains theoretically possible that short-form video engagement itself is associated with better cognitive outcomes through distinct socio-cognitive pathways. These pathways are conceptually divergent, encompassing direct cognitive stimulation via active information seeking, and indirect socio-psychological protection through virtual social interaction and reduced loneliness (25, 49, 50). The conceptual and empirical plausibility of these pathways is supported by prior longitudinal and intervention research, which has documented robust associations between interactive digital media engagement and higher cognitive performance in older cohorts (11, 12, 51–53). Within this context, the multimodal, sensory-rich, and highly heterogeneous content characteristic of short-form video platforms could plausibly elicit similar forms of cognitive engagement. Nevertheless, the cross-sectional design of this study cannot distinguish between these two non-mutually exclusive explanations, and both likely contribute to the observed association to varying degrees. Disentangling the directionality and causal mechanisms underlying this relationship should be a core priority for future longitudinal and interventional research.

Subgroup analyses revealed that the positive association between short-form video use and cognitive function was particularly pronounced among individuals with limited engagement in other cognitive activities (e.g., those who did not exercise, read, or watch TV). This heterogeneous pattern carries both theoretical and methodological implications. Theoretically, it may reflect ceiling effects: for individuals already engaged in multiple cognitive activities, adding short-form video use contributes little additional correlation, whereas for those with few alternatives, digital media use becomes a more salient behavioral marker of cognitive function. Methodologically, this pattern provides critical nuance to the reverse causation discussion: if the association were driven entirely by selection bias—whereby higher-functioning individuals adopt digital media—we might expect the strongest effects among the most cognitively active groups, yet we observe the opposite. Although our cross-sectional data cannot confirm temporal precedence, this heterogeneity suggests that the relationship is more complex than simple reverse causality.

To elucidate the plausible mechanisms linking short-form video use to cognitive performance, it is critical to distinguish between passive consumption and active cognitive engagement. Our findings suggest that the underlying mechanism are not monolithic; rather, they bifurcates depending on usage motives and interaction depth. This divergence aligns with recent longitudinal and meta-analytic evidence (54, 55), likely reflecting the varying levels of cognitive engagement elicited by different motives and interactions. On one hand, passive, leisure-oriented viewing—the predominant pattern in our sample—entails algorithm-driven consumption characterized by shallow processing. This potentially provides insufficient mental stimulation and reinforces habitual over controlled information processing (56–58), explaining its association with poorer executive function. Conversely, goal-directed viewing (driven by informational, educational, or social motives) recruits higher-order cognitive processing and active knowledge acquisition. These goal-directed behaviors inherently engage top-down attentional control, thereby supporting executive networks and working memory stability (57–60). Similarly, socially oriented consumption may potentially support cognitive health by mitigating feelings of isolation and loneliness, whereas passive viewing has been linked to loneliness and depression—both of which are well-documented risk factors for cognitive decline (49, 61, 62). Although our cross-sectional data preclude causal inferences, this empirical pattern aligns with the newly proposed “technological reserve” framework. This framework extends traditional cognitive reserve theory by positing that active, goal-directed digital engagement stimulates cognitive resilience and mitigates decline (63, 64).

On the other hand, the depth of interaction may serve as a parallel mechanism that amplifies cognitive engagement. Unlike passive reception, interactive behaviors are “generative,” requiring the transformation of perceived information into overt actions. For example, sharing requires content evaluation and social-cognitive screening to align with network interests, thereby engaging executive control. Similarly, commenting demands intricate linguistic synthesis and conceptualization, which heavily recruit working memory and semantic comprehension. These complex, multimodal coordination processes represent plausible behavioral stimulants that may theoretically promote neurogenesis and synaptic plasticity among older adults (65–67). Nevertheless, the potential for reverse causality remains a critical caveat, as baseline cognitive proficiency may fundamentally dictate one's capacity for deeper interaction. Active online engagement, unlike passive viewing, imposes substantial cognitive demands; furthermore, the rapid, ongoing evolution of platform features and interfaces necessitates continuous cognitive adaptation. This shifting technological environment poses a formidable barrier to older adults already struggling with digital fluency. Consequently, future longitudinal studies are warranted to dynamically chart these mediating socio-cognitive pathways and untangle the temporal relationship between digital engagement and late-life cognitive health.

### 4.1 Strengths and limitations

This study contributes to the literature by focusing specifically on older adults in rural China, a population frequently underrepresented in digital media research. It provides valuable insights into the adoption and cognitive implications of short-form videos—a medium that has witnessed explosive growth and integration into daily life across diverse demographic groups and regions. By examining the nuances of short-form video use, this study offers a preliminary understanding of how these factors correlate with cognitive health. Finally, the study employed household surveys to gain comprehensive insights into the daily habits and living conditions of rural older adults. This contextual understanding enabled the consideration of a wide range of lifestyle factors potentially influencing cognitive function, allowing for a more precise and robust evaluation of the independent association of short-form video consumption on cognitive function.

Notwithstanding these strengths, several methodological limitations warrant consideration. First, the cross-sectional design precludes causal inferences. The observed associations remain susceptible to reverse causality and self-selection bias. To address this endogeneity, future studies could adopt longitudinal or quasi-experimental designs. Statistical remedies such as proxy variables (e.g., broader digital engagement indicators) or instrumental variables (e.g., exogenous community-level digital infrastructure measures) may also be employed to improve causal identification, as demonstrated in recent empirical applications (68). Second, the single-site recruitment potentially limits the generalizability of our results to other rural contexts with varying digital access, as substantial regional heterogeneity in digital infrastructure and its cognitive implications has been documented among Chinese older adults (13). Third, short-form video metrics relied on self-reported measures, which are susceptible to recall bias and social desirability. A growing body of methodological research, including a major meta-analysis, has documented significant and systematic discrepancies between self-reported and objectively logged digital media consumption (69). Future studies would benefit from integrating objective digital phenotyping or passive logging data to capture real-time media behavior more accurately. Fourth, while the MoCA is a widely accepted screening tool for global cognitive impairment (70), using its subdomain scores to evaluate specific cognitive domains has limited reliability, as the instrument was primarily validated for overall cognitive screening rather than domain-specific diagnosis. Future studies should employ specialized neuropsychological instruments to provide a more nuanced evaluation of individual cognitive domains. Finally, it is worth noting that our data collection was conducted during the post-pandemic period. Although routine public health assessment procedures and community recruitment had fully resumed, the long-term contextual shifts in digital media use patterns induced by the preceding COVID-19 pandemic (71) should be considered when interpreting our findings.

## 5 Conclusions

This study revealed a high prevalence of short-form video use among rural older adults in northern China and its significant association with higher cognitive performance. Furthermore, purposeful engagement—driven by information-seeking, educational, or social motives—alongside active interaction with platform features, was associated with scores across multiple domains. These findings suggest that distinct short-form video usage profiles may serve as meaningful indicators of cognitive status among rural older adults, although reverse causation and residual confounding remain plausible explanations.

## Data Availability

The datasets presented in this study can be found in online repositories. The names of the repository/repositories and accession number(s) can be found in the article/supplementary material.

## Supplementary material

Supplementary file S1 Lifestyle habits and short-form video usage among rural older adults. The questionnaire was specifically designed for this research to investigate the prevalence of short-video engagement and its association with cognitive function.

Supplementary Table S1 Associations between motives, interactive behaviors and cognitive function among short-form video users. Multivariate linear regression models adjusting for covariates revealed short-form video users had higher scores on the MoCA and all five subdomains than non-users did.

Supplementary Table S2 Detailed multivariate linear regression results, model diagnostics, GVIF statistics, HC3 robust standard error analyses, and ordinal logistic sensitivity analyses are provided in Supplementary Table S2.

Supplementary Table S3 Exploratory subgroup analyses for the association between short-form video use and MoCA total score are provided in Supplementary Table S3.

Supplementary Table S4 Associations between motives for short-form video use and cognitive outcomes among short-form video viewers are provided in Supplementary Table S4.

Supplementary Table S5 Associations between interaction variety and cognitive outcomes among short-form video viewers are provided in Supplementary Table S5.

Supplementary Figure S2 Residual diagnostic plots for the multivariate cognition models are shown in Supplementary Figure S2.

Supplementary Figure S3 The forest plot of exploratory subgroup analyses is shown in Supplementary Figure S3.

Supplementary Figure S4 Residual Q-Q plots for the motive-cognition models are shown in Supplementary Figure S4.

Supplementary Figure S5 Residual Q-Q plots for the interaction variety models are shown in Supplementary Figure S5.
