# Supplementary material for: Short-form video usage and cognitive function among rural older adults in northern China: a cross-sectional study
Source: Front Public Health. 2026 Jul 14;14:1873982. doi: 10.3389/fpubh.2026.1873982 (PMC13407642; doi:10.3389/fpubh.2026.1873982)
Supplement: Supplementary file S1 — Lifestyle habits and short-form video usage among rural older adults. The questionnaire was specifically designed for this research to investigate the prevalence of short-video engagement and its association with cognitive function. [file Data_Sheet_1.docx]

**Lifestyle Habits and Short-form Video Usage Among Rural Older Adults.**

Participant ID: _________ Date: _________

**Basic Information**

A1. Biologic Sex: 0. Male 1. Female

A2. Your age: 0. 65-69 1. 70-74 2. 75-79 3. 80 or above

A3. What is your marital status? 0. Never married 1. Married 2. Divorced or separated 3. Widowed

A4. What is your educational level? 0. No formal education 1. Primary school 2. Middle school 3. High school 4. College or above

A5. What is your occupation? 0. Farming 1. Service industry 2. Manual Labor

A6. What is your average monthly disposable income (CNY)? 0. Below 200 1. 201-500 2. 501-1000 3. 1001-2000 4. Above 2000

A7. Number of Cohabitants? _______

**Lifestyle Habits**

B1. Have you smoked at least one cigarette per day for more than one year? 0. Yes 1. No

B2. (if applicable) Number of cigarettes per day: ____ Duration of smoking: ____ Duration since quitting: ____

B3. Have you consumed alcohol for more than one year, with at least 0.1 drink per day? 0. Yes 1. No

B4. (if applicable) Number of drinks per day: ____ Duration of drinking: ____ Duration since quitting: ____

B5. Do you usually engage in farming work or exercise? 0. Yes 1. No

B6. (if applicable) Types of activities: ______ Days per week: ____ Duration per day: ____

B7. Do you usually read books or newspapers? 0. Yes 1. No

B8. (if applicable) Days per week: ____ Duration per day: ____

B9. Do you usually play poker/mahjong/chess (offline)? 0. Yes 1. No

B10. (if applicable) Days per week: ____ Duration per day: ____

B11. Do you usually watch TV? 0. Yes 1. No

B12. (if applicable) Days per week: ____ Duration per day: ____

B13. How is your overall sleep quality? 0. Poor 1. Fair 2. Good

**Medical History**

C1. Do you have any of the following diseases? (Select all that apply) _______________

1. Hypertension 1. Diabetes 2. Stroke 3. Heart disease _____ 4. Other ______

**Short-form video Usage**

D1. Do you usually engage in short-form video (such as Douyin, Kuaishou, Weishi, etc., videos lasting several seconds to a few minutes) 0. Yes 1. No

D2. (if applicable) Days per week: ____ Duration per day: ____ Total duration of usage: ______

D3. What do you mainly use short video apps for? ______ (Select all that apply)

1. Leisure 1. Information seeking 2. Social connection 3. Education 4. Documentation 5. Monetization 6. Other _______

D4. Do you perform the following interactions? ______ (Select all that apply)

1. Like 1. Comment 2. Share/forward 3. Save 4. Follow/subscribe 5. Search actively 6. Tip/Donate 7. Purchase/shop 8. Create/upload 9. Live streaming

### 
