# Supplementary figures and images for: Short-form video usage and cognitive function among rural older adults in northern China: a cross-sectional study
Source: Front Public Health. 2026 Jul 14;14:1873982. doi: 10.3389/fpubh.2026.1873982 (PMC13407642; doi:10.3389/fpubh.2026.1873982)

Associations between short-form video usage and MoCA scores among exploratory subgroups

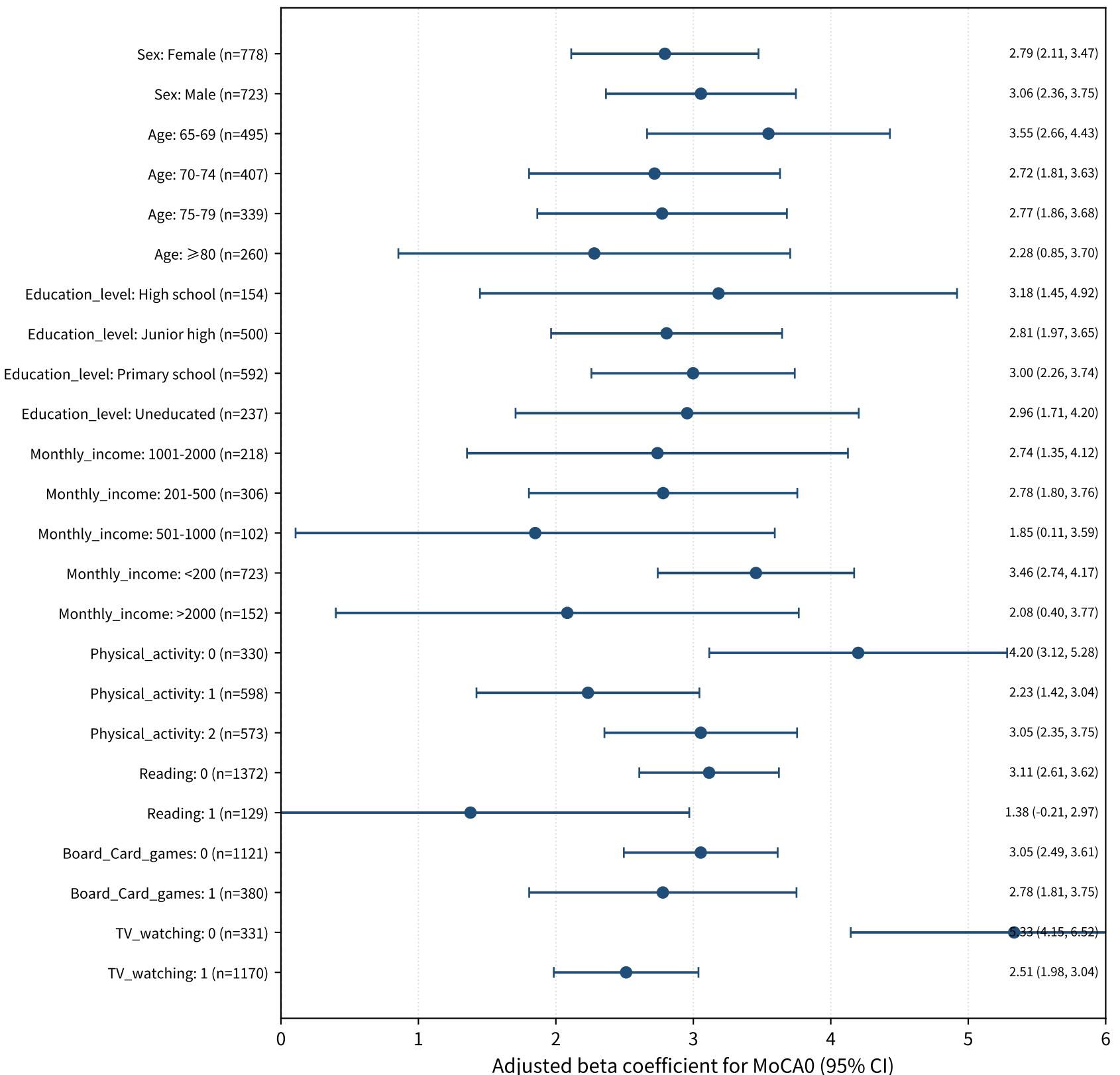

Supplement: Supplementary Table S2 — Detailed multivariate linear regression results, model diagnostics, GVIF statistics, HC3 robust standard error analyses, and ordinal logistic sensitivity analyses are provided in Supplementary Table S2. [file Data_Sheet_2.zip › Supplementary_Figure_S3_Subgroup_forest_plot_MoCA.pdf]

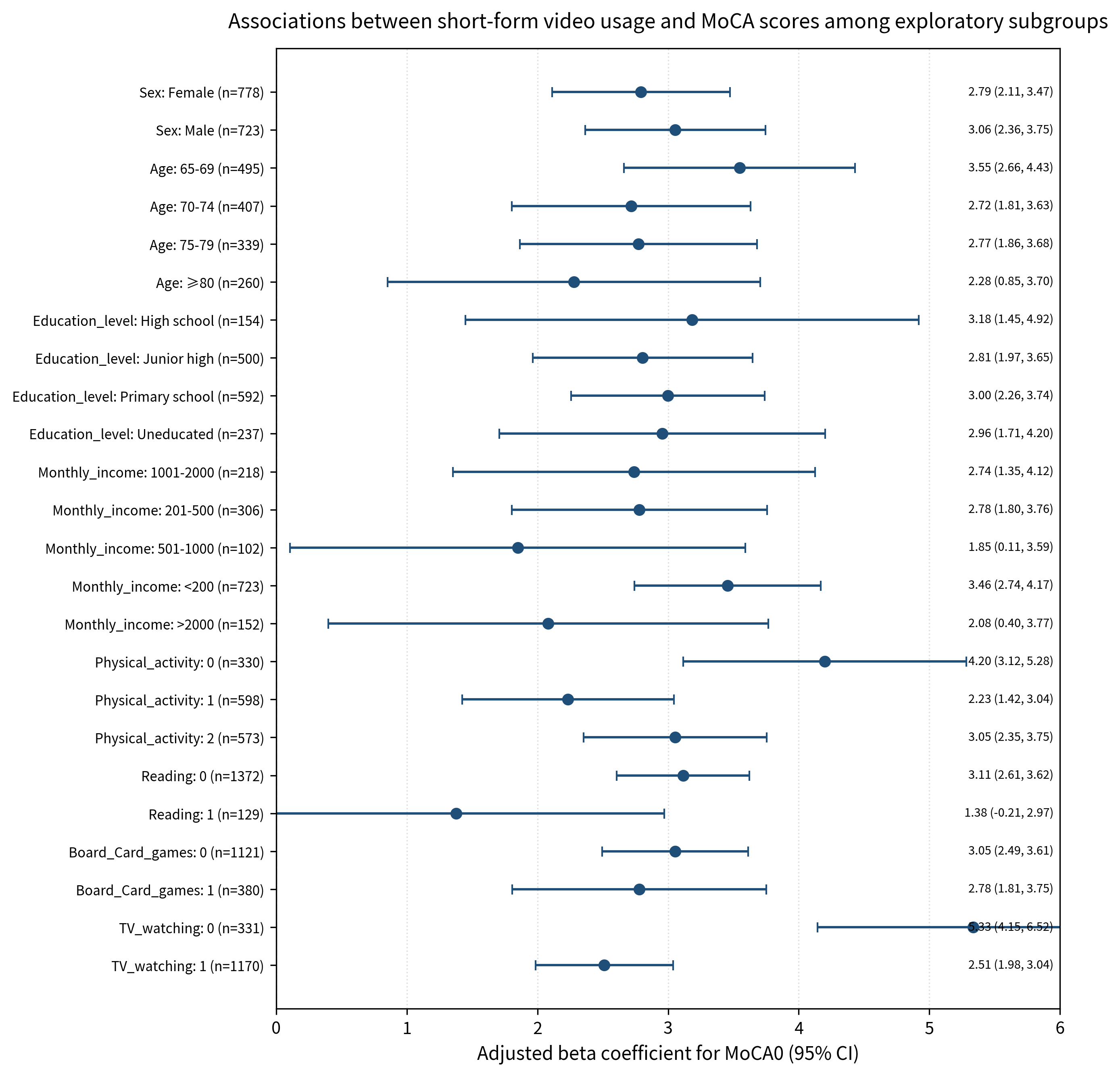

Supplement: Supplementary Table S2 — Detailed multivariate linear regression results, model diagnostics, GVIF statistics, HC3 robust standard error analyses, and ordinal logistic sensitivity analyses are provided in Supplementary Table S2. [file Data_Sheet_2.zip › Supplementary_Figure_S3_Subgroup_forest_plot_MoCA.png]
